# The Time in Therapeutic Range and Bleeding Complications of Warfarin in Different Geographic Regions of Turkey: A Subgroup Analysis of WARFARIN-TR Study

**DOI:** 10.4274/balkanmedj.2016.1617

**Published:** 2017-08-04

**Authors:** Salih Kılıç, Ahmet Çelik, Hüseyin Altuğ Çakmak, Abdülmecit Afşin, Ahmet İlker Tekkeşin, Gönül Açıksarı, Mehmet Erdem Memetoğlu, Fatma Özpamuk Karadeniz, Ekrem Şahan, Mehmet Hayri Alıcı, Yüksel Dereli, Ümit Yaşar Sinan, Mehdi Zoghi

**Affiliations:** 1 Department of Cardiology, Ege University School of Medicine, İzmir, Turkey; 2 Department of Cardiology, Mersin University School of Medicine, Mersin, Turkey; 3 Clinic of Cardiology, Kaçkar State Hospital, Rize, Turkey; 4 Department of Cardiology, İnönü University School of Medicine, Malatya, Turkey; 5 Clinic of Cardiology, Siyami Ersek Training and Research Hospital, İstanbul, Turkey; 6 Clinic of Cardiology, İstinye State Hospital, İstanbul, Turkey; 7 Clinic of Cardiovascular Surgery, Siyami Ersek Training and Research Hospital, İstanbul, Turkey; 8 Clinic of Cardiology, Balıklıgöl State Hospital, Şanlıurfa, Turkey; 9 Clinic of Cardiology, Atatürk Chest Disease and Chest Surgery Training and Research Hospital, Ankara, Turkey; 10 Clinic of Cardiology, 25 Aralık State Hospital, Gaziantep, Turkey; 11 Department of Cardiovascular Surgery, Necmettin Erbakan University Meram School of Medicine, Konya, Turkey; 12 Department of Cardiology, İstanbul University Institute of Cardiology, İstanbul, Turkey

**Keywords:** Warfarin, Awareness, geographical differences

## Abstract

**Background:**

The time in therapeutic range values may vary between different geographical regions of Turkey in patients vitamin K antagonist therapy.

**Aims:**

To evaluate the time in therapeutic range percentages, efficacy, safety and awareness of warfarin according to the different geographical regions in patients who participated in the WARFARIN-TR study (The Awareness, Efficacy, Safety and Time in Therapeutic Range of Warfarin in the Turkish population) in Turkey.

**Study Design:**

Cross-sectional study.

**Methods:**

The WARFARIN-TR study includes 4987 patients using warfarin and involved regular international normalized ratio monitoring between January 1, 2014 and December 31, 2014. Patients attended follow-ups for 12 months. The sample size calculations were analysed according to the density of the regional population and according to Turkish Statistical Institute data. The time in therapeutic range was calculated according to F.R. Roosendaal’s algorithm. Awareness was evaluated based on the patients’ knowledge of the effect of warfarin and food-drug interactions with simple questions developed based on a literature review.

**Results:**

The Turkey-wide time in therapeutic range was reported as 49.5%±22.9 in the WARFARIN-TR study. There were statistically significant differences between regions in terms of time in therapeutic range (p<0.001). The highest rate was reported in the Marmara region (54.99%±20.91) and the lowest was in the South-eastern Anatolia region (41.95±24.15) (p<0.001). Bleeding events were most frequently seen in Eastern Anatolia (41.6%), with major bleeding in the Aegean region (5.11%) and South-eastern Anatolia (5.36%). There were statistically significant differences between the regions in terms of awareness (p<0.001).

**Conclusion:**

Statistically significant differences were observed in terms of the efficacy, safety and awareness of warfarin therapy according to different geographical regions in Turkey.

Warfarin is an effective oral anticoagulant that is used for the prevention of thromboembolic events, especially in patients with atrial fibrillation (AF) and a prosthetic valve. Vitamin K antagonists (VKAs) reduce stroke by 64% compared to a placebo in patients with AF (1,2). The efficacy and safety of warfarin are dependent on maintenance of the international normalized ratio (INR). The target INR values alter according to the reason for warfarin use. The time in therapeutic range (TTR) should be above 70% for optimal efficacy and safety as well as to limit the risk of complications due to warfarin use (3-6). The risk of total mortality and major bleeding increases with irregular follow-up, an insufficient number of INR controls and/or a TTR value below 70% (3,5).

The TTR values may vary between countries in patients VKA therapy (7,8). In our country, the rate of TTR is lower than expected, and differences between geographical regions remain unknown for the time being (9,10,11). However, the ROCKET-AF study reported that TTR rates may vary among geographical regions (12).

In this study, the differences in TTR and their possible causes are examined in patients who participated in the WARFARIN-TR study (The Awareness, Efficacy, Safety and Time in Therapeutic Range of Warfarin in the Turkish Population) and used warfarin regularly for at least 12 months (9). In addition, the efficacy, safety and awareness of warfarin use were evaluated according to the different geographical regions in Turkey.

## MATERIALS AND METHODS

The design, conduct and main results of the WARFARIN-TR study have been presented previously (9). In brief, the WARFARIN-TR study is a multicentre prospective observational study that included 42 centres from 24 cities in seven regions of Turkey. Patients (n=4987, mean age: 60.7±13.5 years, 44.9% male) attended follow-ups for 12 months. Out of the total number of patients, 2124 (42.6%) had a mechanical valve, 1918 (38.4%) had non-valvular AF and 985 (19%) had other conditions as warfarin indications, including chronic pulmonary embolism, ischaemic stroke, deep venous thrombosis, thrombus in any heart chamber, peripheral arterial thrombosis and rheumatic mitral stenosis with AF. The data, including key patient characteristics, treatment, concurrent illnesses and bleeding complications, were recorded.

The study protocol was approved by the local ethics committee. The patients’ data were obtained and recorded during routine clinical follow-up, and the INR values were recorded from the hospital records. Patients who were under 18 years of age, used warfarin inconsistently or did not attend INR monitoring sessions consistently were excluded from the study. The patients’ INR data were extracted for the period January 1, 2014 to December 31, 2014. Patients’ INR values were recorded between each measured INR. Patients with a time between any two measurements of ≥59 days (4.8% of the intervals between two INR measurements) were excluded from the TTR calculation and the study. TTR was calculated as the proportion of days with INR values between the target INR (2.0-3.0 or 2.5-3.5). The safety and efficacy of warfarin therapy are dependent on maintaining the INR with the target 2.0-3.0 for patients with non-valvuler AF and other reason. The INR value target was 3 (2.5-3.5) in patients with a mechanical mitral valve and/or mechanical heart valves in both the aortic and mitral position (13). TTR was calculated according to F.R. Roosendaal’s algorithm with linear interpolation (14).

The awareness of warfarin use was evaluated by simple yes/no questions developed based on a literature review: “Do you know why you use warfarin?” and “Do you know anything about drug-food interactions with warfarin?” were the two questions asked (15). 

Major bleeding was defined as a symptomatic bleeding in a critical organ, transfusion of two or more units of blood or a decrease in haemoglobin level of at least 2 g/L.

In this WARFARIN-TR substudy, the efficacy, safety and awareness of warfarin use were evaluated according to the different geographical regions in Turkey.

### Statistical analysis 

All the data were analysed using SPSS (Statistical Package for the Social Sciences) software for Windows Version 22.0. ANOVA was used to compare numerical variables between groups and for the subgroup comparisons least significant difference test. The relationship between categorical variables was tested by chi-square test to determine important factors of TTR values. General linear models were constructed and a multivariate logistic regression model was built for the bleeding dependent variable. The study sample was selected by stratified analysis according to the density of the regional population (Eastern Anatolia, South-eastern Anatolia, Central Anatolia, the Black Sea, Mediterranean, Aegean and Marmara) and according to Turkish Statistical Institute data (Table 1). P values of <0.05 were considered statistically significant. 

## RESULTS

### Risk scores:

There were statistically significant differences between regions in terms of CHA_2_DS_2_-VASc score, which showed the risk of stroke in patients with non-valvular AF (n=1918) (Table 2). The CHA_2_DS_2_-VASc score was statistically lower in South-eastern and Eastern Anatolia than in other regions. In addition, the scores for Marmara, the Aegean, the Black Sea and Central Anatolian regions were found to be similar and there was no statistically significant difference. A comparison of CHA_2_DS_2_-VASc scores between regions is summarized in Table 3. There were statistically significant differences between regions according to the HAS-BLED score, which indicates the risk of bleeding (Table 2). The bleeding score was reported to be significantly low in the South-eastern Anatolia, East Anatolia and Mediterranean regions, whereas the bleeding score was significantly high in the Black Sea region. The bleeding scores of patients were similar in the Central Anatolia, Aegean and Marmara regions (Table 4).

### Time in therapeutic range:

 Turkey-wide TTR was reported as 49.5%±22.9 in the WARFARIN-TR study. There were statistically significant differences between TTR according to different geographical regions of Turkey (Table 2). The TTR of the Marmara (54.99%±20.91) and Aegean (54.65±24.21) regions was similar and statistically higher than all other regions (p<0.001). Additionally, the TTR of South-eastern Anatolia (41.95±24.15) was close to that of East Anatolia (44.37±23:54) and significantly lower than other regions (p<0.001) (Table 5). Numerous parameters in the multivariate linear regression model were found to be effective on TTR. A low TTR was associated with female patients (compared to male; B=-1.32, p=0.044), a lack of knowledge of warfarin use (compared to good knowledge; B=3403, p=0.012), being uninformed about food-drug interactions (compared to informed; B=-4 807, p=0.001), chronic renal failure (compared to without chronic renal failure; B=-6569, p=0.001) and high risk scores of bleeding. In addition, TTR increased with aging (B=0.126, p=0.001), long duration of warfarin use (B=0.018, p=0.022) and CHA_2_DS_2_-VASc score (B=1.708, p=0.001) (Table 6). Also, the number of INR controls in a year and concomitant antiplatelet therapy were not found to be effective on TTR.

Regional differences were found to be effective on the TTR after purification of significant variables. The TTR was similar to the South-eastern Anatolia region (B=1.528, p=0.3) and significantly higher in the Marmara (B=13.1, p=0.001), Aegean (B=12.2, p=0.001), Mediterranean (B=9:01, p=0.001), Black Sea (B=7.15, p=0.001) and Central Anatolia (B=3.66, p=0.007) regions when the Eastern Anatolia region was considered as a reference.

### Awareness and bleeding:

 The proportion of patients who knew why they were using warfarin was 81.9% in the whole country and only 55% of patients were informed about food and drug interactions. The proportion of patients who knew why they were using warfarin was statistically higher in the Aegean, Anatolia and Central Anatolia (respectively 91.0%, 90.4% and 88.4%) regions; on the other hand, it was similar in the Mediterranean and Marmara regions (respectively 82.7% and 81.1%) and significantly lower in the South-eastern and Black Sea regions (respectively 75.4% and 60.2%). The proportion of patients who were informed about food and drug interactions was statistically higher in the Aegean and Central Anatolian regions (respectively 68.4% and 68.3%), whereas it was lower in the Black Sea and South-eastern Anatolia regions (respectively 30.1% and 31.5%).

There were statistically significant differences between regions in terms of all-cause bleedings and major bleedings (Table 2). Minor bleedings were frequently observed in all regions. All-cause bleedings were significantly higher in the East Anatolia (41.6%) and lower in the South-eastern Anatolia (12.6%) region. Regional differences had an effect on all-cause bleedings and residence in the East Anatolia had a higher risk of bleeding after purification of significant variables (Table 7). Major bleeding was-significantly higher in the Aegean (5.11%) and South-eastern Anatolia (5.36%) and less frequent in the East Anatolia (1.78%), Mediterranean (1.71%) and Marmara (1.95%) regions. Additionally, major bleeding was observed to be nearly three times higher in the Aegean region than in Eastern Anatolia (OR 3.13 95% CI 1:14-8:56 p=0.02) and four times higher in the South-eastern Anatolia region (OR: 4.18 95% CI 1.53-11.4 p=0.005) (Table 8) in the multivariate logistic regression analysis, which evaluated risk factors that may affect major bleedings. In addition, in the same analysis both all-cause bleeding and major bleeding were observed to be higher in patients undergoing concomitant antiplatelet therapy (respectively OR: 1.381 95% CI 1.157-1.648 p=0.001; OR: 1.527 95% CI 1.041-2.239 p=0.030).

## DISCUSSION

The WARFARIN-TR study is important in terms of involving patients that represented the ovarall population of Turkey from all regions and evaluated all-cause warfarin users. Data from the WARFARIN-TR study was evaluated in terms of TTR percentages, efficacy, safety and awareness of warfarin use according to different geographical regions in Turkey. Our study provides national data about TTR for all warfarin users (mechanical valve, non-valvular AF, other conditions) and first time for mechanical valve users. Additionally, it was observed that these patients were the same as other cases. In our study we showed statistically significant differences according to the TTR between different geographical regions of Turkey for the first time and geographical differences were an independent risk factor for TTR. To the best of our knowledge, the present study is the first in published data.

Statistically significant differences were reported between regions according to the elapsed time within the effective range in published data despite the widespread use of warfarin (7). Numerous studies that evaluated warfarin usage have noted that there are differences between countries and regions in terms of TTR (7,12).

Previous studies in Turkey noted low TTR values in this country. The AFTER study, which included 2242 patients with at least one AF attack from 17 different centres in Turkey, reported non-valvular AF (NVAF) and effective INR levels as 78% and 41.3%, respectively (10). In the WATER (Warfarin in Therapeutic Range) study, the mean TTR value was 42.3%±18 in 572 patients with AF (71% NVAF) during 22-month follow-up (11). Although the WARFARIN-TR study’s percentage of TTR is far from the effective range, it was higher than the other published data in our country. Moreover, the TTR appears to be quite low when compared to the data of other countries (7,8). In a single-centre study, TTR levels were not associated with socio-economic status and co-morbid diseases in Turkey (16). The socio-economic status of patients was not evaluated in our study. However, TTR was lower in regions with low socio-economic status and higher in regions with high socio-economic status. Thus, TTR may be affected by socio-economic status in the present study. Ethnic and cultural differences were also apparent between regions in Turkey, which may also affect-the TTR (17,18).

The TTR values were higher in Italy (69.5%) and Spain (64.9%), where patients were followed by special anticoagulation clinics, than in general clinics in the US (58.1%), Canada (62.8%) and France (59.3%). In a similar study, which assessed a one-year INR follow-up of patients in specific (n=233) and general clinics (n=148) in our country, the mean TTR was significantly higher in the specific clinic group (19). Although the outcomes of our study and other studies in Turkey were consistent with the general polyclinic results of this study, specific polyclinic results were significantly higher. Specific clinics may overcome this problem in regions of Turkey with low TTR.

Awareness is the most prominent factor for effective drug therapy in patients. Low TTR outcomes in our study and other studies in Turkey could be explained by a lack of knowledge of warfarin use in nearly one-fifth of patients and drug-food interactions in nearly half of the patients countrywide. Additionally, the high rate of knowledge about drug-food interactions in regions with a high TTR supports this finding. In multivariate analysis, the presence of awareness has a constant impact on TTR. In addition, according to cohort studies, with aging, long-term use of warfarin TTR increases and our results were consistent with these results (20,21). 

Bleeding rates, an indicator of reliability, were found to be higher in the overall country and minor bleeding was observed more frequently. Although bleeding risk scores were significantly different between regions, all patients had a low bleeding profile. The statistically significant differences between the regions and ongoing observation of these differences in the multivariate analysis in terms of both all-cause bleeding and major bleeding showed that geographical differences have a marked influence on bleeding in Turkey, just like TTR. However, socio-economic, ethnic and cultural differences may also have an effect on bleeding among regions. The combination of VKA and antiplatelet therapy is associated with both increased all-cause bleeding and major bleeding compared with VKA monotherapy. In multivariate analysis, we showed that both all-cause bleeding and major bleeding are significantly increased in a combination of VKA and antiplatelet therapy.

The main limitation of our study is that we did not record embolic complications at follow-up.

In conclusion, statistically significant differences were reported in terms of the efficacy, safety and awareness of warfarin therapy according to different geographical regions in Turkey. However, these differences may have emerged due to other reasons such as socio-economic, cultural and ethnic differences that were not assessed in the present study. Therefore, further researches should be performed with larger study groups to achieve more accurate results.

## Figures and Tables

**Table 1:**
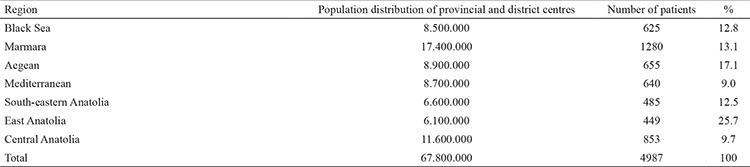
The population distribution in regions in the WARFARIN-TR study

**Table 2:**
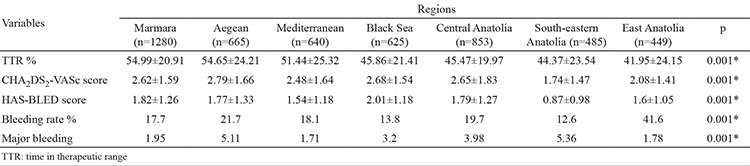
The distribution of time in therapeutic range values. bleeding rates and risk scores according to geographical region

**Table 3:**
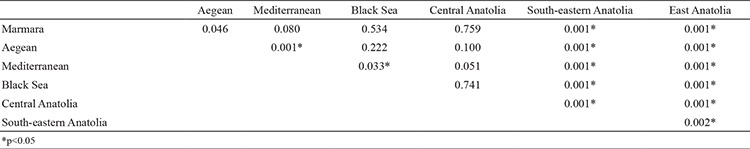
Comparison of CHA2DS2VASc scores between regions

**Table 4:**
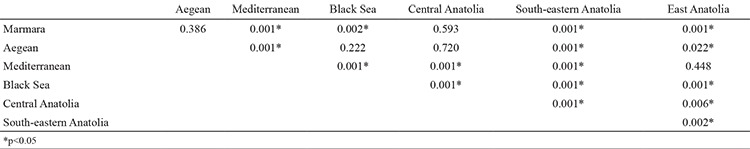
Comparison of HAS-BLED scores between regions

**Table 5:**
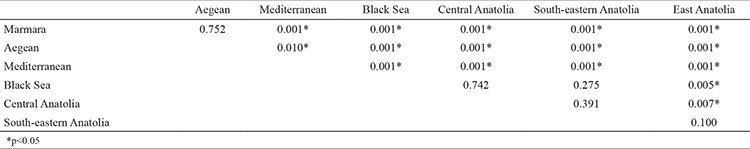
Comparison of time in therapeutic range rates according to regions

**Table 6:**
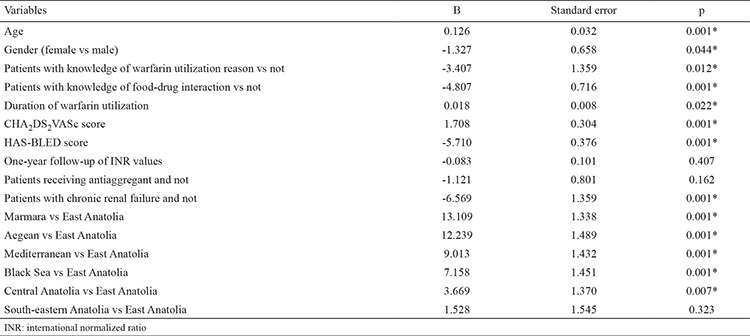
Results of general linear model to determine important factors for time in therapeutic range values

**Table 7:**
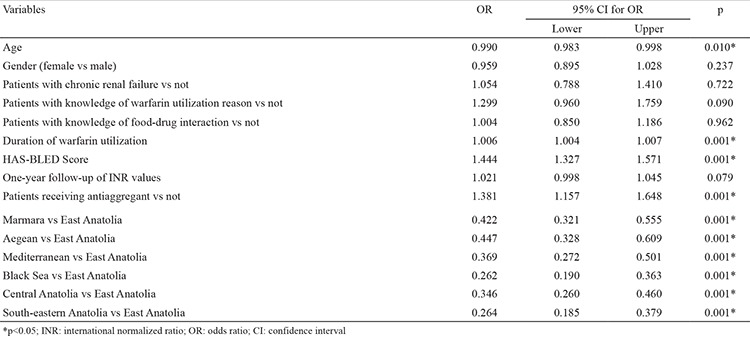
Multivariate logistic regression results to determine important factors of bleeding

**Table 8:**
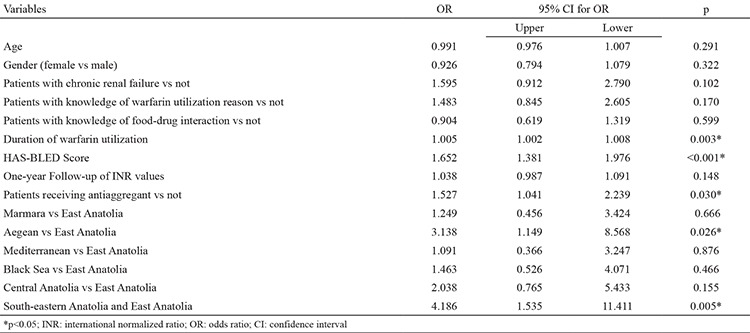
Multivariate logistic regression results to determine important factors of major bleeding
